# Mental Health Research in the Syrian Humanitarian Crisis

**DOI:** 10.3389/fpubh.2014.00044

**Published:** 2014-05-16

**Authors:** Hussam Jefee-Bahloul, Kaveh Khoshnood

**Affiliations:** ^1^Department of Psychiatry, Yale School of Medicine, New Haven, CT, USA; ^2^Yale School of Public Health, New Haven, CT, USA

**Keywords:** mental health, Syrian population, refugees, humanitarian, academic research

In areas of armed conflicts, efforts to provide mental health services for refugees and internally displaced populations (IDPs) generally lack measures of effectiveness, and the gap between research and practice is significant (1). The Syrian Crisis has been described by the United Nations High Commissioner for Refugees (UNHCR) as “the great tragedy of this century” (2). The UNHCR reports at least 24 agencies currently providing mental health and psychological support to Syrian refugees (3). The actual interventions provided vary considerably among the agencies, which complicates the implementation of academic research and mental health services in humanitarian settings (4). This is further challenged by the limited academic publications on the matters of Syrian refugees’ mental health beyond basic needs assessment (3, 5–7). In an effort to standardize mental health interventions in humanitarian settings, the Inter-Agency Standing Committee (IASC) had published standardized guidelines for provision of mental health services, but had not addressed the role of academic research (8). Creating a research agenda for mental health in the less-resourceful settings has been emphasized (9). However, there are barriers to establish academic research in emergency humanitarian settings. Those barriers have been identified before (8), and we will mention few examples here in relevance to the Syrian scenario.

The research conducted in conflict settings is often designed and executed by “foreign” institutions outside the area of the conflict or disaster (8), and the Syrian Crisis is no exception (5, 6, 10). This is likely to affect the efficacy and sustainability of interventions. Incorporating local institutions and humanitarian workers in designing and conducting the research would likely foster a sense of ownership in the locals and ensure sustainability. For instance, thousands of educated Syrian youth have sought refuge in the neighboring countries since the beginning of the conflict (11). Incorporating this educated population as research assistants and cultural brokers in mental health research projects would enhance ownership, sustainability, and efficacy of such projects. Another obstacle for academic research is reaching the non-refugee; i.e., displaced populations. The internally displaced living within the battlefields inside Syria, is an example (10). Additionally, mental health research in refugee camps across the borders requires availability of basic needs such as shelter, food, water, and basic medical services which are not always secured, or stable (12), and can interrupt research efforts. Hence, initiating needs assessment, intervention implementation, and effectiveness studies in the relatively more stable areas such as Turkey (13) can allow for manageable pilot research projects. In the future, those studies can be replicated and tested in less stable areas.

In a recent study (currently in press and detailed elsewhere), one of us (Hussam Jefee-Bahloul) and colleagues surveyed a sample of Syrian refugees in a busy refugee primary care clinic in Kilis Turkey (6). The assessment used a standardized and validated tool that can reflect psychological stress. In brief, a significant number of refugees (41%) met the cut off criteria for needing further psychological assessment; however, less than a half had a perceived need to see a mental health specialist, and less so was open to mental health services provided via technology (telepsychiatry). The example of telepsychiatry here is relevant to the topic of this article as it represents an example of mental health innovative interventions that require testing in humanitarian settings. Conducting a sufficient basic needs assessment in a very busy primary care refugee clinic had mandated administration of a simple, time-efficient, and non-threatening questionnaire rather than standard psychological stress test batteries. Recruitment process was received with mixed reactions by the refugees; either desperation to seek help or suspiciousness of the foreign workers (even though our data collection was done by an Arabic speaking Arab-American medical student). Also, subjects of the study were hesitant to cooperate given the stigma surrounding mental illness in the Syrian culture. Nevertheless, this kind of needs assessment studies can be replicated and used as cornerstones for interventions effectiveness studies in the unstable conflict areas of Syria.

Here thereafter, a philosophical gap lies between the academic “perfectionistic” views on research and the “practical” perspectives of humanitarian workers (e.g., the focus of humanitarian workers on immediate short-term-outcomes rather than long-term-outcomes) (8). This gap is justified by the emergency context of these situations. Refugees fleeing conflict areas may exhibit acute symptoms of psychological stress that require immediate attention by the humanitarian workers. The application of academic research in these acute settings may not guarantee immediate effective remedies for people under duress. Humanitarian workers may also have concerns that application of research designs will interrupt the flow of humanitarian work. This can explain the hesitance to incorporate research in conflict settings. On the other hand, the expansion of this gap will prevent field-based humanitarian interventions from catching up with evidence-based medicine. Hence, the need to bridge this gap and address these valid concerns has never been more imperative. Incorporating thoughtfully designed interventions and standardized outcome measurements into the routine humanitarian protocols and training manuals may help solve this problem. It is worth mentioning that some academic projects had successfully incorporated academic research into humanitarian settings in the past (14–19) and more is hoped to be accomplished on mental health of those affected by the Syrian Crisis.

Finally, the schism between the academic and the humanitarian platforms and audiences adds to the observed gap. The academic literature and the humanitarian platforms rarely merge. Some academic journals have designated certain issues to humanitarian causes (4), however, this does not guarantee delivery of this information to the target audience, i.e., humanitarian organizations, workers, and policy makers. Unifying the academic and humanitarian platforms, can allow audiences from both fields to interact, share experience, and collaborate on much needed mental health research in areas of conflict.

## Conflict of Interest Statement

The authors declare that the research was conducted in the absence of any commercial or financial relationships that could be construed as a potential conflict of interest.
